# Data of multiple regressions analysis between selected biomarkers related to glutamate excitotoxicity and oxidative stress in Saudi autistic patients

**DOI:** 10.1016/j.dib.2016.02.025

**Published:** 2016-02-15

**Authors:** Afaf El-Ansary

**Affiliations:** a Biochemistry Department, Science College, King Saud University, Saudi Arabia; b Autism Research and Treatment Center, Riyadh, Saudi Arabia; c Medicinal Chemistry Department, National Research Centre, Dokki, Guiza, Egypt

**Keywords:** Autism, Multiple Regression Analysis, Glutamate excitotoxicity, Oxidative stress, Detoxification, Glutathione status

## Abstract

This work demonstrates data of multiple regression analysis between nine biomarkers related to glutamate excitotoxicity and impaired detoxification as two mechanisms recently recorded as autism phenotypes. The presented data was obtained by measuring a panel of markers in 20 autistic patients aged 3–15 years and 20 age and gender matching healthy controls. Levels of GSH, glutathione status (GSH/GSSG), glutathione reductase (GR), glutathione-s-transferase (GST), thioredoxin (Trx), thioredoxin reductase (TrxR) and peroxidoxins (Prxs I and III), glutamate, glutamine, glutamate/glutamine ratio glutamate dehydrogenase (GDH) in plasma and mercury (Hg) in red blood cells were determined in both groups. In Multiple regression analysis, *R*^2^ values which describe the proportion or percentage of variance in the dependent variable attributed to the variance in the independent variables together were calculated. Moreover, *β* coefficients values which show the direction either positive or negative and the contribution of the independent variable relative to the other independent variables in explaining the variation of the dependent variable were determined. A panel of inter-related markers was recorded. This paper contains data related to and supporting research articles currently published entitled “Mechanism of nitrogen metabolism-related parameters and enzyme activities in the pathophysiology of autism” [1], “Novel metabolic biomarkers related to sulfur-dependent detoxification pathways in autistic patients of Saudi Arabia [2], and “A key role for an impaired detoxification mechanism in the etiology and severity of autism spectrum disorders” [3].

**Specifications table**

**Table:** 

Subject area	*Clinical Chemistry*
More specific subject area	*Biomarkers of autism*
Type of data	*Tables and figure*
How data was acquired	*ELISA Technique analysis of blood samples*
Data format	*Analyzed data*
Experimental factors	*Blood was collected in test tubes containing heparin as anticoagulant. The samples were separated by centrifugation at* 3000 rpm *and* 25 °C *for* 10 *min. The plasma was removed and frozen at* −80 °C *until analyzed.*
Experimental features	*The selected biomarkers were measured in plasma samples of autistics and control participants*
Data source location	*Riyadh, Saudi Arabia, Autism Research & Treatment Center clinic*
Data accessibility	*All data is with this article*

## **Value of the data**

• Identification of novel biomarker for neurological disorders will help to increase the quality of life of affected individuals, by providing sensitive and selective clinical correlates for the early diagnosis.
• These markers can provide insights into disease mechanisms that can be used to identify therapeutic targets and to develop efficacious compounds to target them.
• Due to the complexity of the brain, a single marker is not sufficient to have enough diagnostic power and thus it is important to combine panel of markers to improve diagnosis accuracy [5].
• Among different combination approaches, the multiple regressions are easy to compute and interpret the relationship between different recorded markers. It is a common statistical technique to assess the relationships among two or more independent variables and their correlation with a dependent variable.
• Screening of this panel of markers in newborns at risk for neuro-developmental disease (e.g. autism) can help in the early diagnosis and intervention.

## 1 Data

**Fig. 1:**
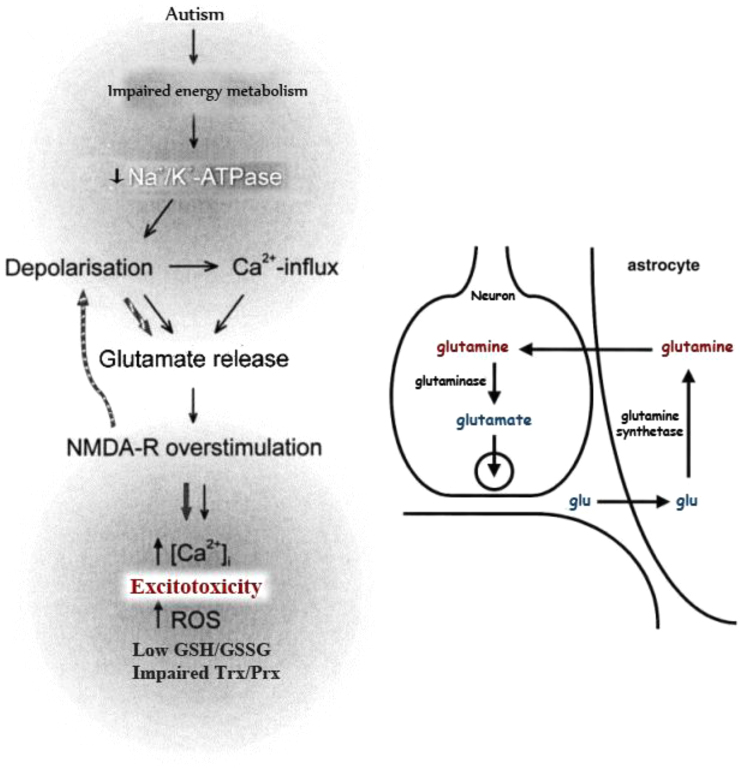
Illustrated relationship between the associated glutamate excitotoxicity, oxidative stress and impaired detoxification markers in autistic patients.

Table 1 demonstrates the significant variations of the measured parameters in autistic patients compared to healthy controls. *R*^2^ values of the multiple regression analysis for glutamate, glutamine and glutamate/glutamine ratio as three dependent variables show that almost 100% of the changes of these variables could be easily explained and associated by the changes in oxidative stress and detoxification related parameters in autistic patients but not in age and gender matching control participants (Table 2, Table 3, Table 4, Table 5). The relationship between the panels of associated biomarkers is illustrated in Fig. 1.

## 2 Experimental design, materials and methods

The local Ethical Committee of the Faculty of Medicine, King Saud University, Riyadh, Saudi Arabia, approved this study (Approval number is 11/2890/IRB). In addition, an informed written consent of participation for this study was signed by the parents or the legal guardians of the investigated subjects, according to the Helsinki principles. All subjects enrolled in the study (20 autistic children and 20 control males) had filled the informed consent. They were enrolled through the ART Center (Autism Research & Treatment Center) clinic in King Khalid University Hospital in Riyadh. The ART Center clinic population consisted of children diagnosed on the autism spectrum disorder (ASD). The diagnosis of ASD was confirmed in all subjects using the Autism Diagnostic Interview-Revised (ADI-R) and the Autism Diagnostic Observation Schedule (ADOS) and Developmental, dimensional diagnostic interview (3DI). The mean of age of all autistic children participated in the study were between 7±4 years old. All were simplex cases. All were negative for fragile×gene study. The control group recruited from pediatric clinic at king Saud medical city in Riyadh with mean age 7±4 years old. Subjects were excluded from the investigation if they had dysmorphic features, or diagnosis of fragile X or other serious neurological (e.g., seizures), psychiatric (e.g., bipolar disorder) or known medical conditions. All participants were screened via parental interview for current and past physical illness. Children with known endocrine, cardiovascular, pulmonary, liver, kidney or other medical disease were excluded from the study.

### 2.1 Blood samples

After an overnight fast, patients underwent blood sampling; 10 ml blood samples were collected on ice from both groups in test tubes containing heparin as anticoagulant. The samples were separated by centrifugation at 3000 rpm and 25 °C for 10 min. The plasma was removed and frozen at −80 °C until analyzed.

## 3 Biochemical analyses

Glutamate and glutamine levels were assessed using an HPLC method. For measurement of glutamate dehydrogenase activity, a commercial kit (Randox Laboratories Ltd., Crumlin, Co. Antrim, UK) was used. Thioredoxin 1 (Trx 1) together with the peroxiredoxins (Prxs I and III) was assessed using ELISA kits, a product of northwest company. Thioredoxin reductase (TR) activity was measured using commercially available kit (Biovision, USA). Measurement of reduced GSH, total glutathione and GSH/GSSG ratio were assayed based on the glutathione recycling system by 5,5-dithio-bis (2-nitrobenzoic acid) (DTNB) and glutathione reductase. The glutathione-s-transferase (GST) activity was assessed using (Biovision, USA) assay kit. The concentration of inorganic mercury (Hg) in red blood cells was determined using a flameless atomic-absorption instrument.

### 3.1 Statistical analyses

Statistical Program for Social Sciences (SPSS) (SPSS Inc., Chicago, IL, USA) was used for all analyses. Data were expressed as mean±SD. All statistical comparisons were made by means of Student’s *t*-test. *P*<0.05 was considered significant. Multiple regression analysis was used to find the correlation between the selected parameters using SPSS program [4]. In this analysis *R*^2^ describes the proportion or percentage of variance in the dependent variable explained by the variance in the independent variables together which sometimes called the predictor variables. An *R*^2^ of 1.00 indicates that 100% of the variation in the dependent variable is explained by the independent variables. Conversely, an *R*^2^ of 0.0 indicates the absence of variation in the dependent variable due to the independent variables. In this work as the variables are not in the same unit of measures, a standardized regression coefficient, beta (*β*), was used. The *β* coefficients values show the direction either positive or negative and the contribution of the independent variable relative to the other independent variables in explaining the variation of the dependent variable. *R*^2^ and (*β*) coefficient provide most of what we need to interpret our multiple regression data. Stepwise multiple regression analyses were performed using glutamate, glutamine and glutamate/glutamine ratio as three dependent variables and Trx1, Trxreductase, Prx I&III, GSH/GSSG, glutathione-s-transferase and mercury as independent variables.

## Figures and Tables

**Table 1:** Mean±SD of the measured chemicals in plasma or red blood cells of patients with autism compared with age-matched controls.

Parameter	Group	*N*	Mean±SD	Percent change	*P* value

Glutamic (µmol/l)	Control	20	111.91±4.51	100.00	0.001
Autistic	20	152.80±6.47	136.54
Glutamine (µmol/l)	Control	20	241.82±12.93	100.00	0.001
Autistic	20	111.34±5.69	46.04
Glutamic/Glutamine Ratio	Control	20	0.46±0.03	100.00	0.001
Autistic	20	1.37±0.06	296.18
Glutamate dehydrogenase (GLDH) (U/l)	Control	20	1.71±0.47	100.00	0.001
Autistic	20	0.93±0.36	54.22
Thioredoxin I (ng/ml)	Control	20	44.71±7.43	100.00	0.001
Autistic	20	74.70±9.04	167.09
Thioredoxin reductase (mU/ml)	Control	20	1.83±0.52	100.00	0.001
Autistic	20	3.31±1.11	180.87
Peroxiredoxin I (ng/ml)	Control	20	19.58±4.76	100.00	0.001
Autistic	20	34.56±8.32	176.55
Peroxiredoxin III (ng/ml)	Control	20	24.30±2.69	100.00	0.001
Autistic	20	43.05±5.86	177.16
GSH/GSSG	Control	20	26.07±5.03	100.00	0.001
Autistic	20	8.03±2.46	30.79
Glutathione-s-transferase (µmol/min/ml)	Control	20	0.69±0.20	100.00	0.001
Autistic	20	0.41±0.12	59.26
Mercury (µg/L)	Control	20	4.64±0.68	100.00	0.001
Autistic	20	6.93±0.74	149.40

**Table 2:** Multiple regression using stepwise method for glutamic acid (µmol/l) as a dependent variable in autistic group.

Predictor variable	Beta	*P* value	Adjusted *R*^2^	Model	
*F* value	*P* value
Glutamine (µmol/l)	0.302	0.001	0.964	352.514	0.001
Glutamic/glutamine ratio	92.604	0.001
Peroxiredoxin 1 level (ng/ml)	−0.273	0.011

**Table 3:** Multiple regression using stepwise method for glutamine (µmol/l) as a dependent variable in autistic group.

Predictor variable	Beta	*P* value	Adjusted *R*^2^ square	Model	
*F* value	*P* value
Glutamic (µmol/l)	0.969	0.001	0.990	1243.754	0.001
Glutamic/glutamine ratio	−177.627	0.001
Thioredoxin 1 level (ng/ml)	−0.274	0.044

**Table 4:** Multiple regression using stepwise method for glutamic/glutamine ratio as a dependent variable in autistic group.

Predictor variable	Beta	*P* value	Adjusted *R*^2^	Model	
*F* value	*P* value
Glutamic (µmol/l)	0.006	0.001	0.995	1938.823	0.001
Glutamine (µmol/l)	−0.005	0.001
Thioredoxin reductase activity (mU/ml)	0.013	0.038
GSH/GSSG	−0.003	0.018

**Table 5:** Multiple regression using stepwise method for glutamic (µmol/l) as a dependent variable in control group.

Predictor variable	Beta	*P* value	Adjusted *R*^2^	Model	
*F* value	*P* value
Glutamic/glutamine ratio	222.768	0.001	0.996	2481.60	0.001
Glutamine (µmol/l)	0.445	0.001
