# Supplementary material for: Data of multiple regressions analysis between selected biomarkers related to glutamate excitotoxicity and oxidative stress in Saudi autistic patients
Source: Data Brief. 2016 Feb 15;7:111–6. doi: 10.1016/j.dib.2016.02.025 (PMC4764897; doi:10.1016/j.dib.2016.02.025)
Supplement: Supplementary file 1 — Supplementary material [file mmc1.doc]

The Author declared that no conflict of interest
